# ICAM-1 expression on chondrocytes in rheumatoid arthritis: induction by synovial cytokines

**DOI:** 10.1155/S0962935192000140

**Published:** 1992

**Authors:** M. E. Davies, H. Sharma, R. Pigott

**Affiliations:** 1 Strangeways Research Laboratory Worts Causeway Cambridge CB1 4RN UK; 2 British Biotechnology Ltd Watlington Road Cowley Oxford OX4 5LY UK

## Abstract

The intercellular adhesion molecule-1 (ICAM-1) was found by immunostaining chondrocytes in cartilage from three patients with rheumatoid arthritis. Expression of ICAM-1 was restricted to chondrocytes in areas of erodedcartilage adjacent to the invading synovial tissue. Toluidine blue staining of these areas demonstrated severe depletion of the cartilage extracellular matrix. In areas of undamaged cartilage there was no ICAM-1 expression. Since ICAM-1 is not constitutively expressed on normal human articular cartilage, but could be induced in vitro by exogenous IL-1α, TNFα and IFNγ or by co-culturing cartilage with inflammatory rheumatoid synovium, we conclude that the induction of ICAM-1 on rheumatoid chondrocytes results from the synergistic action of a variety of cytokines produced by the inflammatory cells of the invading pannus.


Mediators of Inflammation 1, 71–74 (1992)

### ICAM-1 expression on chondrocytes in rheumatoid arthritis: induction by synovial cytokines

M. E. Davies,[1,CA] H. Sharma[1] and R. Pigott[2]

[1]Strangeways Research Laboratory, Worts Causeway, Cambridge CB1 4RN, UK;
[2]British Biotechnology Ltd, Watlington Road, Cowley, Oxford OX4 5LY, UK.

[CA] Corresponding Author

**THE intercellular adhesion molecule-1 (ICAM-1) was found by immunostaining chondrocytes in cartilage from three patients with rheumatoid arthritis. Expression of ICAM-1 was restricted to chondrocytes in areas of eroded cartilage adjacent to the invading synovial tissue. Toluidine blue staining of these areas demonstrated severe depletion of the cartilage extracellular matrix. In areas of undamaged cartilage there was no ICAM-1 expression. Since ICAM-1 is not constitutively expressed on normal human articular cartilage, but could be induced *in vitro* by exogenous IL-1α, TNFα and IFN$_{\gamma}$ or by co-culturing cartilage with inflammatory rheumatoid synovium, we conclude that the induction of ICAM-1 on rheumatoid chondrocytes results from the synergistic action of a variety of cytokines produced by the inflammatory cells of the invading pannus.**

**Key words:** Articular cartilage, Chondrocytes, Cytokines, ICAM-1, Rheumatoid arthritis

### Introduction

Rheumatoid arthritis (RA) is an autoimmune inflammatory disease characterized by infiltration of the cartilage matrix by inflamed synovial tissue which ultimately destroys the joint. Although the mechanism initiating the process of inflammatory invasion is unknown, it is presumed to involve an active and self-perpetuating immune response as indicated by the presence of large numbers of activated T-cells, macrophages, fibroblasts and plasma cells.[1,2] This cellular environment is a rich source of cytokines e.g., IL-1, TNFα, PDGF, IL-6, GM-CSF, IFN$_{\gamma}$[2] which may function in a variety of processes, including induction of activation markers on both synovial cells[2] and chondrocytes,[3,4] the indigenous cell of the cartilage.

One such activation marker is HLA Class II, expression of which is a prerequisite for antigen presentation and the subsequent initiation of an immune response. Class II has been demonstrated on RA chondrocytes[5] possibly due to the synergistic action of synovial IFN$_{\gamma}$, TNFα and GM-CSF,[2] and evidence of the ability of chondrocytes to present autoantigens is also available.[1]

Maintenance of expression of Class II and efficient antigen presentation is now thought to require the aid of cell adhesion molecules such as ICAM-1.[2,6] Since we have already shown ICAM-1 to be inducible by IL-1 on normal human articular cartilage *in vitro*,[7] it would obviously be of interest to know whether this potentially important molecule is expressed under the pathophysiological conditions of the inflamed joint.

In the present study we have investigated the *in vivo* expression of ICAM-1 on human rheumatoid cartilage chondrocytes and have demonstrated the requirement for cytokines in its induction. Our findings provide additional evidence for the involvement of cytokines and chondrocyte–inflammatory cell interactions in the pathogenesis of rheumatoid arthritis.

### Materials and Methods

Rheumatoid cartilage was obtained following hip and knee replacement surgery from three patients, a 40 year-old ♂, 59 year-old ♀ and a 48 year-old ♂, all suffering from RA as defined by the American Rheumatism Association.[8] The patients had been treated with non-steroidal anti-inflammatory drugs. The extent of cartilage damage in the rheumatoid joints was assessed as far as possible macroscopically and two to three adjacent cartilage explants were removed from each different area as soon as possible after surgery. The explants were frozen in O.C.T. embedding medium (Miles Diagnostics) and stored at −70°C until used for immunohistology. Normal articular cartilage was obtained postoperatively from selected patients having no recorded history of inflammatory joint disease. In the experiments described here the patients were both male, aged 61 and 80, and receiving no medication. The cartilage explants were used within 6 h and were either frozen immediately in O.C.T. and stored at −70°C to provide *ex vivo* control tissue, or were cultured under standard conditions as described previously[7] in DMEM +5% foetal calf

© 1992 Rapid Communications of Oxford Ltd serum (i) in the presence of different concentrations of exogenous human recombinant IL-1α, TNFα or $IFN_\gamma$ for 4 h–6 days or (ii) in contact with several different samples of human inflammatory rheumatoid synovium using the method described previously.[4]

Immunohistology was performed on 6–9 μm frozen sections of the cartilage as already described in detail elsewhere[3] except that, in the present study, the primary antibody was a mouse anti-human ICAM-1, prepared and characterized at British Biotechnology[7] and the secondary antibody was biotinylated goat anti-mouse Ig/streptavidin-HRP complex (Sigma Poole, Dorset, UK). After development of the colour reaction using diaminobenzidine as substrate, the sections were mounted and viewed with a Nikon diaphot microscope fitted with epifluorescence.

### Results

*ICAM-1 expression on rheumatoid cartilage:* By indirect immunolocalization we demonstrated staining for ICAM-1 on chondrocytes in diseased cartilage removed from knee and hip joints from three patients with rheumatoid arthritis. From each patient two to three explants removed from areas of badly damaged, medium or undamaged cartilage were examined. In all three patients expression of ICAM-1 was observed on chondrocytes in areas of damaged cartilage adjacent to the invading synovium (Fig. 1). Although cells were sparse, the majority showed positive staining. These areas of cartilage appeared eroded by both macroscopical and microscopical examination, and toluidine blue staining showed extensive depletion of the extracellular matrix proteoglycans. In the samples examined so far chondrocytes in the subarticular and midzones of the cartilage did not express ICAM-1.

Some positive staining was observed at restricted sites in the synovial tissue, indicating ICAM-1 expression by a small proportion of the inflammatory cells.

Cartilage explants removed from areas distant from the invading pannus appeared undamaged and there was no evidence of ICAM-1 expression by the chondrocytes.

*Experimental induction of ICAM-1 on normal cartilage*

*(i) By in vitro culture with cytokines:* We have shown previously that ICAM-1 is not expressed constitutively on normal human cartilage chondrocytes, but can be induced during *in vitro* culture with IL-1α.[7]

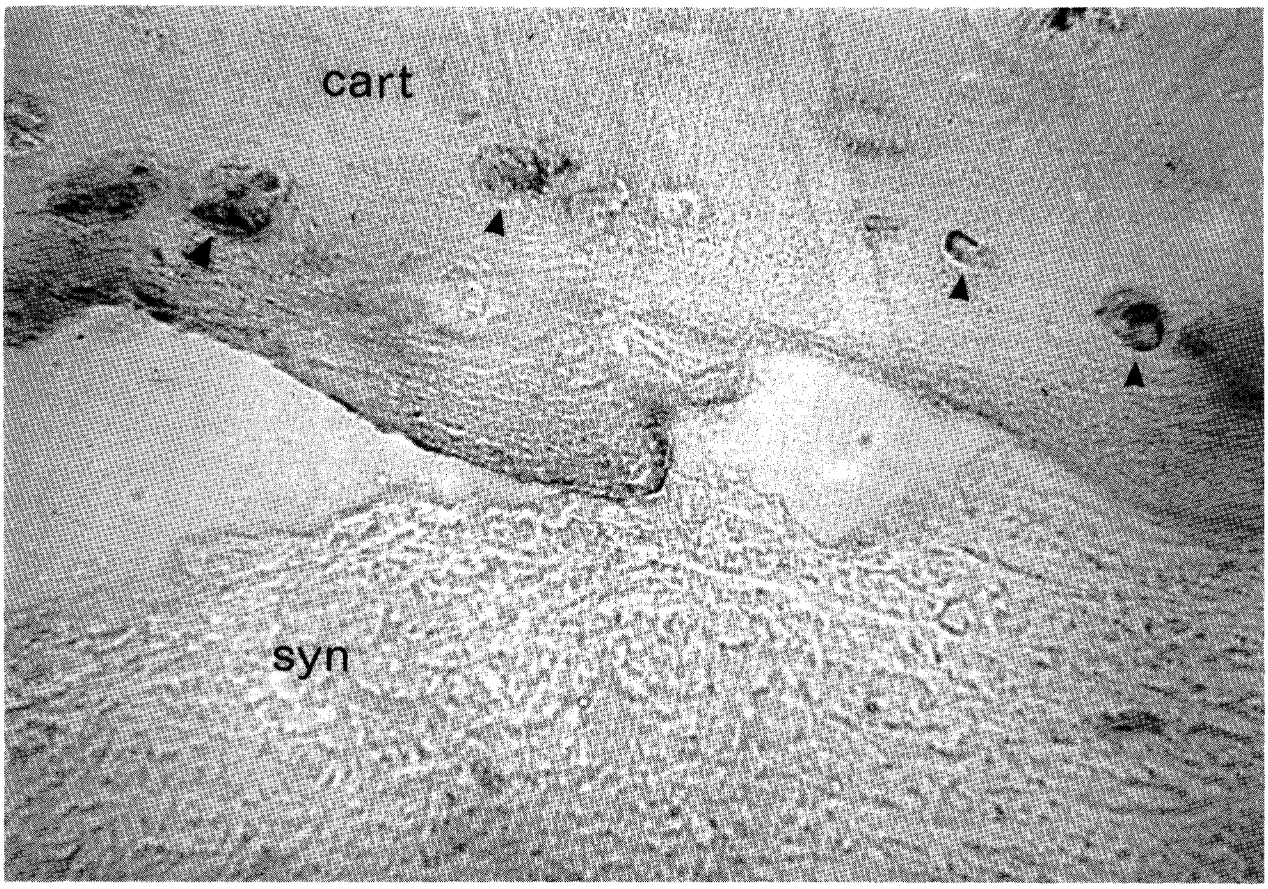

FIG. 1. Expression of ICAM-1 on chondrocytes (arrowed) in human rheumatoid arthritic cartilage.

**Table 1.** Dose-response and time-course of induction of ICAM-1 by cytokines on chondrocytes in normal human cartilage*

| | Time in culture | | |
|---|---|---|---|
| | 4 h | 24 h | 6 days |
| Dose of IL-1α (ng/ml) | | | |
| 0.1 | – | + | + |
| 1.0 | – | ++ | ++ |
| 2.0 | – | +++ | ++ |
| Dose of TNFα (ng/ml) | | | |
| 1.0 | – | + | + |
| 5.0 | – | + | + |
| 10.0 | – | ++ | + |
| Dose of $IFN_γ$ (ng/ml) | | | |
| 0.1 | – | ± | + |
| 1.0 | – | + | + |
| 10.0 | – | ++ | +++ |

*No cells staining (–); majority (>80%) of cells staining weakly(±), moderately (+), strongly (++), very intensely (+++).

In the present study we have shown that like IL-1α both TNFα (dose range 1.0–10.0 ng $ml^{-1}$) and $IFN_γ$ (dose range 0.1–10.0 ng $ml^{-1}$) had no effect at 4 h but were able to induce ICAM-1 expression comparable with IL-1α during culture for 24 h–6 days. The dose-response and time-course experiments were done twice on both normal cartilage samples and the combined results are presented in Table 1.

*(ii) By co-culture with rheumatoid synovium:* When normal human articular cartilage was co-cultured in close contact with minced inflammatory rheumatoid synovium strong expression of ICAM-1 was induced on the chondrocytes (Fig. 2). The most intense staining was seen on cells in close proximity to the synovial tissue. Moving away from the synovium the intensity of staining gradually reduced as the distance increased, until all cells were negative for ICAM-1.

Marked expression of the adhesion molecule was also demonstrated on clusters of cells within the synovial tissue.

This experiment has been repeated on three separate occasions using different samples and has yielded the same results each time.

### Discussion

Our observation that chondrocytes in diseased human rheumatoid cartilage express the adhesion

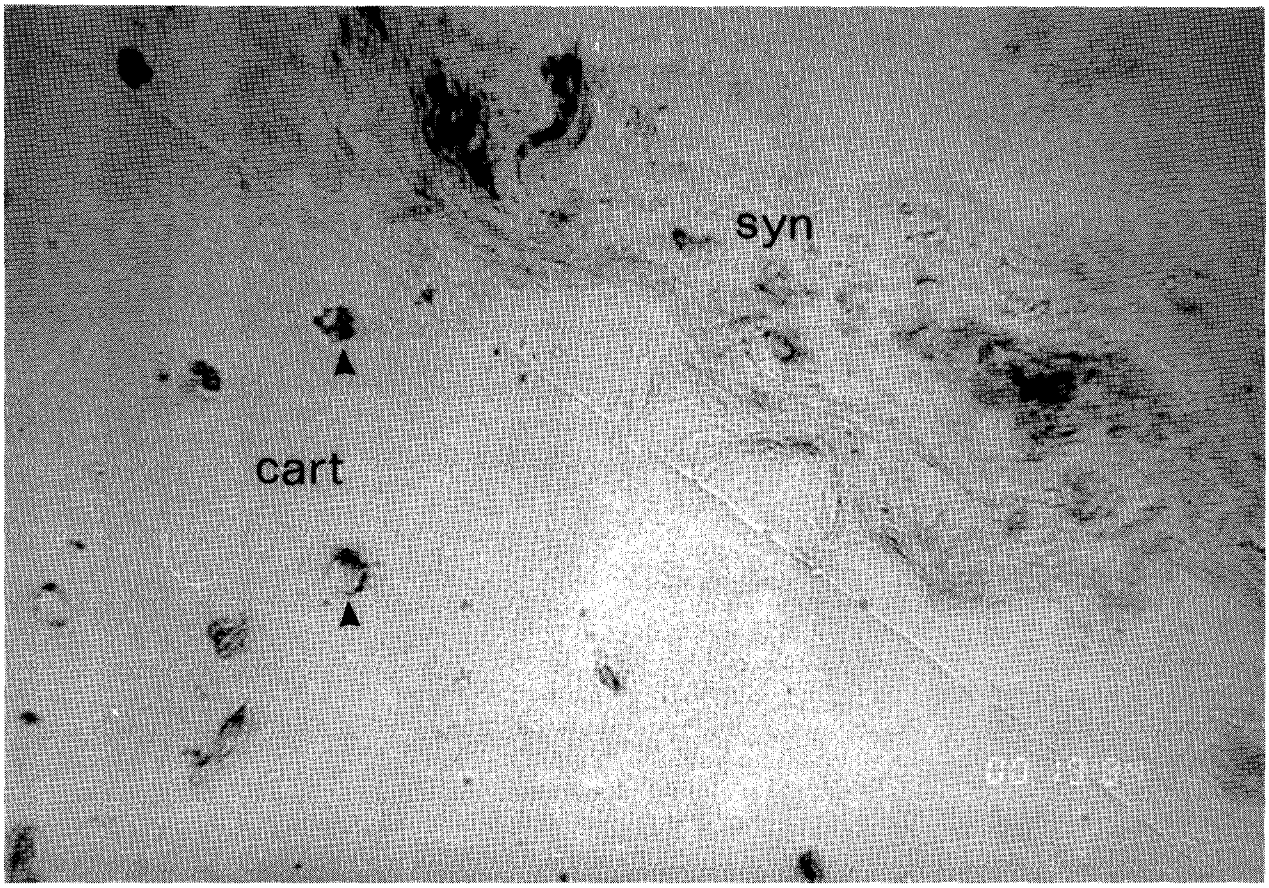

FIG. 2. Co-culture of normal human cartilage with inflammatory rheumatoid synovium. Note the expression of ICAM-1 on chondrocytes (arrows) and on synovial cells. syn, synovium; cart, articular cartilage.

molecule, ICAM-1 has revealed both a new marker of cytokine activation and possibly a novel role for the chondrocyte in cell-cell interactions.

We have shown that chondrocytes in normal, undamaged cartilage do not express ICAM-1[7] but require activation by pro-inflammatory cytokines such as IL-1$\alpha$, TNF$\alpha$ or IFN$_\gamma$, a finding consistent with reports of ICAM-1 expression in other cell types.[6,9]

In rheumatoid cartilage, expression of the adhesion molecule was found to be restricted to areas of damaged and proteoglycan-depleted cartilage at the junction with the overlying pannus. This observation suggests a very localized action of inflammatory mediators presumably originating from the synovial infiltrate. Our co-culture experiment confirmed that inflammatory synovium is a very effective inducer of ICAM-1 on chondrocytes, due to its production of adequate amounts of the necessary activating cytokines e.g. IL-1$\alpha$ and $\beta$, TNF$\alpha$ and IFN$_\gamma$.[4,10,11] Although we have demonstrated the effectiveness of these individual cytokines *in vitro*, the situation is more complicated in the inflammatory microenvironment in RA, where these mediators will be acting perhaps synergistically and certainly in concert with a variety of growth factors (TGF $\beta$, PDGF, IGF1 and GM-CSF) as well as with inhibitors or antagonists.[12] As far as we know, this is the first reported observation of ICAM-1 expression by chondrocytes under pathophysiological conditions. Further experiments are now in progress to assess (i) whether this is a general phenomenon of RA cartilage and (ii) how expression of the adhesion molecule is regulated in this complex microenvironment. The co-culture would seem to provide a suitable *in vitro* model for investigation of the mechanisms of chondrocyte activation, expression of adhesion molecules, and their roles in the regulation of cartilage extracellular matrix turnover.

### References

1. Alsalameh S, Mollenhauer J, Hain N, *et al.* Cellular immune response toward human articular chondrocytes. T-cell reactivities against chondrocytes and fibroblast membranes in destructive joint diseases. *Arthritis Rheum* 1990; **33:** 1477–1486.
2. Brennan FM, Field M, Chu CQ, *et al.* Cytokine expression in rheumatoid arthritis. *Br J Rheumatol* 1991; **30 (Suppl.1):** 76–80.
3. Dingle JT, Davies ME, Mativi BY, *et al.* Immunological identification of interleukin-1 activated chondrocytes. *Ann Rheum Dis* 1990; **49:** 889–892.
4. Davies ME, Horner A, Dingle JT. Immunorecognition of chondrocytes in articular cartilage activated by synovial interleukin-1. *Conn Tiss Res* 1991; **25:** 243–249.
5. Jahn B, Burmester GR, Schmid H, *et al.* Changes in cell surface antigen expression on human articular chondrocytes induced by gamma-interferon: induction of Ia antigens. *Arthritis Rheum* 1987; **30:** 64–74.
6. Allard SA. Cell adhesion. *Br J Rheumatol* 1990; **29:** 137–139.
7. Davies ME, Dingle JT, Pigott R, *et al.* Expression of intercellular adhesion molecule 1 (ICAM-1) on human articular cartilage chondrocytes. *Conn Tiss Res* 1991; **26:** 207–216.
8. Arnett FC, Edworthy SM, Bloch DA, *et al.* The American Rheumatism Association 1987 revised criteria for the classification of rheumatoid arthritis. *Arthritis Rheum* 1988; **31:** 315–324.
9. Chin JE, Winterrowd GE, Krzesicki RF, *et al.* Role of cytokines in inflammatory synovitis. *Arthritis Rheum* 1990; **33:** 1776–1786.
10. Miyasaka N, Sato K, Goto M, *et al.* Augmented interleukin-1 production and HLA-DR expression in the synovium of rheumatoid arthritis patients: possible involvement in joint destruction. *Arthritis Rheum* 1988; **31:** 480–486.
11. Lipski PE, Davis LS, Cush JJ, *et al.* The role of cytokines in the pathogenesis of rheumatoid arthritis. *Springer Semin Immunopathol* 1989; **11:** 123–162.
12. Arend WP, Dayer J-M. Cytokines and cytokine inhibitors or antagonists in rheumatoid arthritis. *Arthritis Rheum* 1990; **33:** 305–315.

ACKNOWLEDGEMENT. We thank Dr JT Dingle for support and encouragement.

**Received 25 November 1991;**

**accepted with revision 11 December 1991.**
